# The Golgin GMAP210/TRIP11 Anchors IFT20 to the Golgi Complex

**DOI:** 10.1371/journal.pgen.1000315

**Published:** 2008-12-26

**Authors:** John A. Follit, Jovenal T. San Agustin, Fenghui Xu, Julie A. Jonassen, Rajeev Samtani, Cecilia W. Lo, Gregory J. Pazour

**Affiliations:** 1 Program in Molecular Medicine, University of Massachusetts Medical School, Worcester, Massachusetts, United States of America; 2 Department of Physiology, University of Massachusetts Medical School, Worcester, Massachusetts, United States of America; 3 Laboratory of Developmental Biology, National Heart Lung Blood Institute, National Institutes of Health, Bethesda, Maryland, United States of America; Washington University School of Medicine, United States of America

## Abstract

Eukaryotic cells often use proteins localized to the ciliary membrane to monitor the extracellular environment. The mechanism by which proteins are sorted, specifically to this subdomain of the plasma membrane, is almost completely unknown. Previously, we showed that the IFT20 subunit of the intraflagellar transport particle is localized to the Golgi complex, in addition to the cilium and centrosome, and hypothesized that the Golgi pool of IFT20 plays a role in sorting proteins to the ciliary membrane. Here, we show that IFT20 is anchored to the Golgi complex by the golgin protein GMAP210/Trip11. Mice lacking GMAP210 die at birth with a pleiotropic phenotype that includes growth restriction, ventricular septal defects of the heart, omphalocele, and lung hypoplasia. Cells lacking GMAP210 have normal Golgi structure, but IFT20 is no longer localized to this organelle. GMAP210 is not absolutely required for ciliary assembly, but cilia on GMAP210 mutant cells are shorter than normal and have reduced amounts of the membrane protein polycystin-2 localized to them. This work suggests that GMAP210 and IFT20 function together at the Golgi in the sorting or transport of proteins destined for the ciliary membrane.

## Author Summary

The primary cilium is a sensory organelle used by cells to monitor the extracellular environment. In mouse, severe defects in primary cilia lead to embryonic lethality while less severe defects cause a pleiotrophic phenotype that includes cystic kidney disease, retinal degeneration, obesity, and hydrocephaly, among others. The sensory functions of cilia rely on proteins localized to the ciliary membrane, which is continuous with the plasma membrane of the cell. Cells have the ability to specifically localize proteins to the ciliary membrane to the exclusion of the rest of the plasma membrane. Little is known about how this is accomplished. In prior work, we showed that the ciliary assembly protein IFT20 is localized to the Golgi complex, in addition to the cilium, and we proposed that it is involved in sorting or transport of membrane proteins to the cilium. In this work, we show that IFT20 is anchored to the Golgi complex by the golgin GMAP210. Mice defective in GMAP210 die at birth with lung and heart defects. Cells from these animals have ciliary defects, suggesting that IFT20 and GMAP210 function together at the Golgi complex in the trafficking of ciliary membrane proteins.

## Introduction

Most vertebrate cells have a non-motile primary cilium projecting from their surface [1],[2]. Defects in these organelles lead to a wide range of developmental disorders and diseases ranging from embryonic lethality in severe cases to polycystic kidney disease and retinal degeneration with less extreme alleles. These non-motile primary cilia are thought to be sensors of the extracellular environment. A number of receptors and channels have been localized to the ciliary membrane including the opsin photoreceptors of the vertebrate retina, the odorant receptors of the olfactory system, the SSTR3 isoform of the somatostatin receptor [3], smoothened and patched, transmembrane receptors in the hedgehog signaling pathway [4],[5], the PDGFRα isoform of the platelet derived growth factor receptor [6], and the polycystins and fibrocystin, products of the human polycystic kidney disease genes [7]–[9].

Little is known about how the ciliary membrane is assembled and maintained despite the fact that this membrane is vitally important for the sensory functions of cilia. While the ciliary membrane is continuous with the plasma membrane of the cell it is a separate domain with a unique complement of proteins localized to it [10]. The mechanism separating the ciliary membrane domain from the rest of the apical plasma membrane is likely to involve a membrane-cytoskeletal complex called the ciliary necklace [11]. The proteins that make up these complexes are as yet unknown, but probably help form the diffusional barrier separating the two zones. There is also a zone of condensed lipid at the base of the cilium that may contribute to the barrier [12]. Membranous vesicles containing ciliary membrane proteins appear to dock on the plasma membrane just outside of the cilium [13],[14]. Recent studies are beginning to identify the protein machinery required for trafficking to the ciliary membrane. In *C. elegans*, progress has been made in identifying proteins required for transport of membrane proteins into the dendrite, which is a prerequisite step for ciliary membrane targeting in this organism, but proteins required specifically at the cilium are still unknown [15]. In vertebrates, Rab8 appears to regulate the transport of membrane proteins to the cilium as expression of dominant negative Rab8 causes opsin-containing vesicles to accumulate at the base of the cilium [16] and also prevents the formation of cilia in cultured cells [17]. Defects in proteins required for polarization of mammalian cells such as FAPP2 [12], Crumbs3-CLPI [18], annexin-13, and syntaxin-3 [19] also perturb ciliogenesis, but whether these are acting directly on transport of ciliary proteins or indirectly in the formation of the apical domain is not known [12]. Smoothened transport in mammalian cells requires beta-arrestin [20] although this is not required for transport of polycystin-2 in *C. elegans* [15].

Intraflagellar transport (IFT) is responsible for assembling the non-membrane portions of the cilium (reviewed in [21],[22]) but its role in movement of membrane proteins is not clear. During IFT, large complexes, composed of ∼20 proteins are transported along the ciliary microtubules under the membrane [23],[24]. The complexes are thought to carry proteins from their site of synthesis in the cell body to sites of assembly in the cilium. The IFT particles traffic along the microtubule axoneme just under the flagellar membrane and probably interact with the membrane [25],[26]. The nature of the connection between the ciliary membrane and the particle is not obvious as none of the known IFT particle proteins have any predicted transmembrane domains [27]. In *C. elegans*, membrane channels move in cilia at rates that are comparable to those of IFT, suggesting that IFT moves proteins within the ciliary membrane [28] and in *Chlamydomonas*, movement of a membrane associated kinase into the cilium requires IFT [29]. Levels of the transmembrane protein, polycystin-2, are elevated in cilia when the IFT88 subunit is mutated in *C. elegans* [30], mouse [7], and *Chlamydomonas* [31] suggesting that IFT88 may be more important for removing polycystin-2 from the cilium than inserting it into the cilium.

We previously showed that one of the IFT particle proteins, IFT20, is localized to the Golgi complex as well as to the cilium and the peri-basal body pool. We hypothesized that IFT20 plays a role in the sorting or transport of membrane proteins processed through the Golgi complex and destined for the ciliary membrane. This idea was based on the observation that IFT20 moved between the Golgi and ciliary compartments and the demonstration that partial reduction of IFT20 by RNAi reduced the level of the membrane protein polycystin-2 in cilia [32]. In this work we sought to further our understanding of the function of the Golgi-associated pool of IFT20 by identifying proteins that interact with IFT20 at the Golgi complex. To do this, we immunoprecipitated an IFT20-containing complex from mouse kidney cells and used mass spectrometry (MS) to identify one of the subunits as a golgin known as GMAP210 or TRIP11. This peripheral membrane protein was previously shown to be localized to the Golgi complex by a number of groups [33]. Beyond localization to the Golgi complex, there is little agreement in the literature about the function of this protein in mammals and it has been proposed to play roles ranging from regulating gene expression, controlling Golgi structure, and polarized secretion. To understand the *in vivo* function of GMAP210, we generated a GMAP210 mutant mouse. The mutant mice are viable until birth, when they die from a pleiotrophic phenotype that includes growth retardation and lung and heart defects. Cells derived from these animals do not have structural defects in their Golgi complexes indicating that this protein is not required for Golgi organization. However, IFT20 is displaced from the Golgi complex in mutant cells indicating that GMAP210 anchors IFT20 to the Golgi membrane. In addition, the mutant cells have slightly shorter cilia and have significantly less polycystin-2 in these cilia. This suggests that GMAP210 functions with IFT20 in the trafficking of proteins to the ciliary membrane.

## Results

### Identification of IFT20 Interacting Proteins

**Figure 1:**
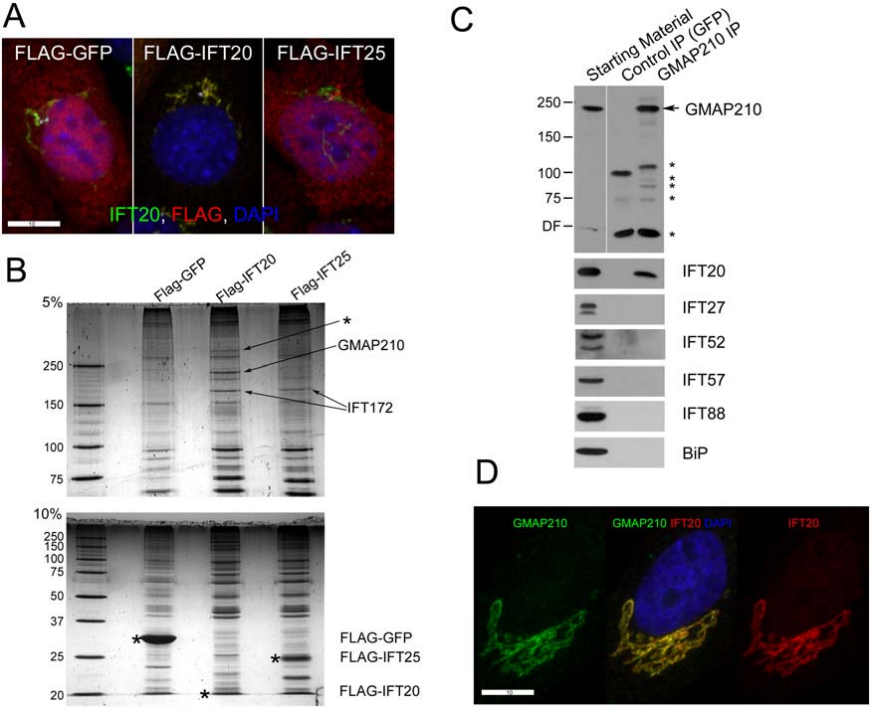
Identification of GMAP210 as an IFT20 binding protein. A. Stable mouse kidney cells lines expressing FLAG-tagged GFP, IFT20, and IFT25 were generated, fixed and stained with DAPI (blue) and antibodies to IFT20 (green) and FLAG (red). Scale bar is 10 µm. B. FLAG IPs from these lines were analyzed by silver stain after SDS-PAGE. The bait proteins are marked with * on the 10% (lower) gel. The bands marked with arrows were analyzed by MS. The large band (*) was not identified. C. Inverse IP. Human RPE cells were IPed with antibodies to GFP and GMAP210 (monoclonal antibody clone 15) and analyzed by western blotting. The GMAP210 antibody precipitated GMAP210 and IFT20 but not any of the other IFT proteins or the negative control protein BiP even though all were present in the starting material. * mark proteins introduced in the IP. D. GMAP210 and IFT20 extensively colocalize in human RPE cells. GMAP210 (green) was detected by monoclonal Ab clone 15. IFT20 (red), DAPI (blue). Scale bar is 10 µm.

IFT20 is the only IFT particle protein known to be associated with the Golgi complex [32]. The identification of proteins that interact with IFT20 at the Golgi membrane is likely to yield new information about the function of IFT20. To this end, we generated stable mouse kidney cell lines expressing FLAG-tagged IFT20 and as controls, FLAG-tagged IFT25 and FLAG-tagged GFP (Figure 1A). IFT25 is a small IFT complex B subunit that is not Golgi associated (Follit et al., in preparation). FLAG-IFT20 localizes predominantly to the Golgi complex, whereas FLAG-IFT25 localizes to the cilium and basal body region as well as the cell body. FLAG-GFP is found in the cell body and is not enriched at either the cilium or Golgi complex. To identify candidate proteins that potentially interact with FLAG-tagged proteins, FLAG-tagged proteins were immunoprecipitated (IPed) from cell lysates using FLAG antibody coupled to agarose, fractionated by SDS-PAGE and the gels silver stained (Figure 1B). Proteins found in all three lanes are background proteins that non-specifically bound to the resin whereas proteins found in the IFT20 and IFT25 extracts are likely to be IFT complex B proteins. This appears to be the case since the ∼200 kD band found in both the IFT20 and IFT25 lanes was identified by mass spectrometry (MS) as IFT172. Two bands were identified in the IFT20 extract but not in either of the controls suggesting that these are IFT20 interacting proteins that are not part of complex B. We were not able to identify the larger band (indicated by an asterisk), but MS identified the smaller band as a golgin protein known in mammals as Thyroid Hormone Receptor Interacting Protein 11 (TRIP11) [34], Golgi Microtubule Associated Protein 210 (GMAP210) [35], and Clonal Evolution Related Protein (CEV14) [36]. The yeast orthologue is known as RUD3p [37].

To verify the interaction between IFT20 and GMAP210, we used a monoclonal antibody against GMAP210 (Clone 15, BD Transduction Laboratories) to perform inverse IPs. This antibody recognizes a single protein in extracts made from human cells (Figure 1C, starting material) but does not recognize the mouse orthologue. Extracts of human retinal pigmented epithelial (RPE) cells were IPed using the GMAP210 monoclonal Ab and a GFP monoclonal Ab (JL-8, Clontech) as a negative control. The GMAP210 Ab but not the GFP Ab precipitated IFT20 (Figure 1C). The IP extracts also were probed with our collection of antibodies directed against mouse IFT proteins that also recognize the human orthologues. Even though all of these proteins were present in the extract, only IFT20 was precipitated by the GMAP210 antibody (Figure 1C). This corroborates the identification of GMAP210 as an IFT20-binding protein and indicates that IFT20 and GMAP210 interact independently of IFT complex B. Furthermore, IFT20 and GMAP210 extensively co-localize at the Golgi complex as would be expected for interacting proteins (Figure 1D).

### Identification of the IFT20 Binding Site on GMAP210

**Figure 2:**
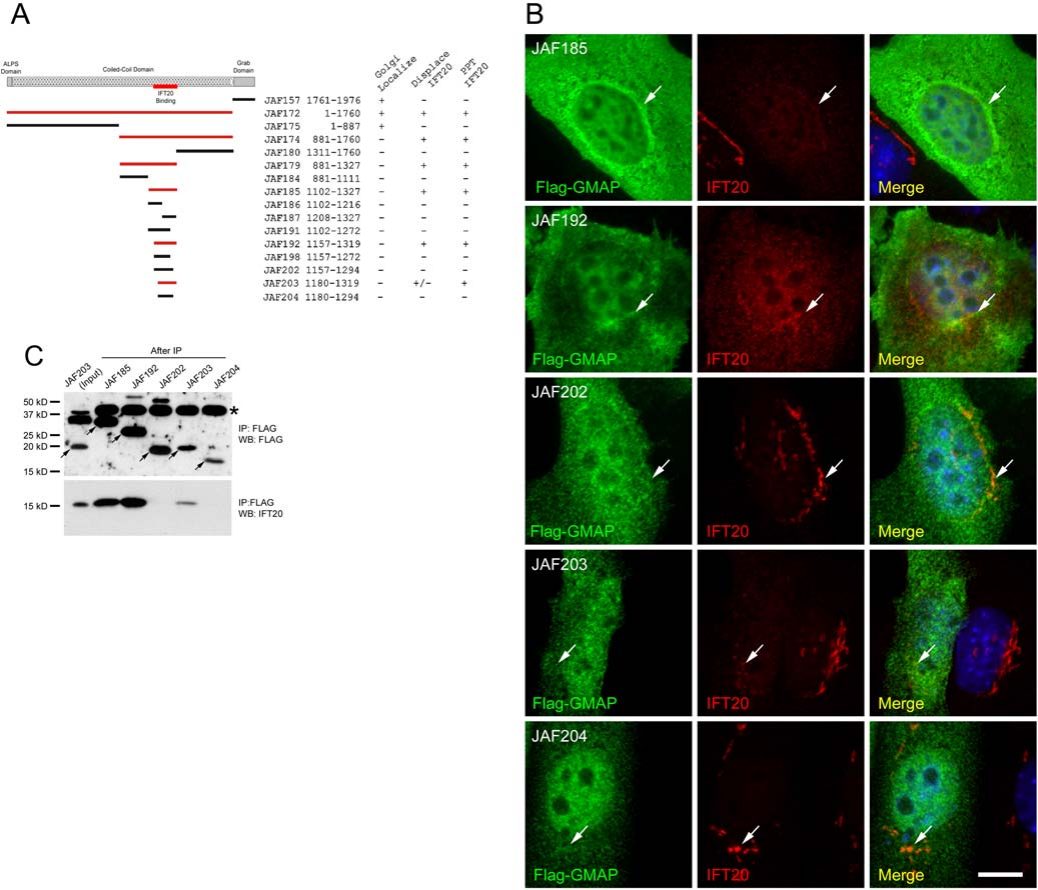
Identification of the IFT20 binding site on GMAP210. A. Schematic map of GMAP210 and summary of the data. Fusion proteins that bind IFT20 are drawn red while those that did not are black. The numbers following the plasmid names are the residues of GMAP210 in the construct. The behavior of the fusion proteins is summarized on the right. B. Selected images of cells expressing the fusion proteins illustrating the main points from Figure 2A. GMAP210 fragments were detected with FLAG antibody staining (green), endogenous IFT20 with our antibody (red) and nuclei with DAPI (blue). Note that fragments of GMAP210 containing the IFT20 binding site (JAF185, JAF192, JAF203) did not bind to the Golgi on their own but did displace IFT20 from the Golgi complex. Scale bar is 10 µm. C. Co-IP of IFT20 by selected GMAP210 fragments to illustrate main points from Figure 2A. The top panel shows the GMAP210 fusion proteins after IP with FLAG. The bottom panel show these IPs probed with the IFT20 antibody to determine if the fragment of GMAP210 is capable of binding IFT20. The smallest fragment capable of binding IFT20 is encoded by JAF203 although the fragment encoded JAF192 is more effective. The band marked with * is a cross reacting protein IPed and detected by the FLAG antibody. Arrows mark the GMAP210-FLAG fusion proteins. JAF203 (Input) is the starting material to illustrate the level of IFT20 in these cells while the remaining lanes are after IP.

To map the IFT20 binding site on GMAP210, we tested whether IPing FLAG-tagged fragments of GMAP210 also brought down IFT20 and whether these FLAG-GMAP210 fragments could displace IFT20 from the Golgi apparatus by competing with native GMAP210 for binding to IFT20. The identity of the Golgi-targeting sequence within GMAP210 is controversial, with the targeting sequence variably being located to the N- or C-terminal ends of the protein [33] so we also examined the cellular distribution of our FLAG-tagged GMAP210 constructs. Data are graphically displayed in Figure 2A, key examples of IF and IP that document the IFT20 binding site are shown in Figures 2B and 2C while IF data supporting the Golgi localization are shown in Figure S1A. Initially, we expressed GMAP210 as two fragments split at the junction between the coiled-coil and Grab domains (JAF157 and JAF172, Figure 2A). Both fragments localized to the Golgi-complex, although the N-terminal fragment (JAF172) also was found in the cytoplasm (Figure S1A). The C-terminal fragment (JAF157) did not affect the localization of native IFT20 or bring down IFT20 in an IP. The N-terminal fragment (JAF172) precipitated IFT20 and partially displaced IFT20 from the Golgi complex indicating that it contains an IFT20 binding site. We then split the JAF172 fragment into two smaller fragments. The N-terminal JAF175 fragment partially localized to the Golgi complex indicating that there are Golgi-targeting domains at both the N- and C-terminal ends of the protein (Figure S1A). Thus our results explain the apparent discrepancy in the literature [33], which can be ascribed to a non-systematic analysis of the protein in previous studies [34],[37],[38]. We did not precisely map the Golgi-binding domain at the N-terminus, but it is likely to involve the ALPS domain that has recently been shown to bind curved membranes [39]. The JAF174 fragment displaced IFT20 from the Golgi and was able to IP IFT20 indicating that it contained the IFT20 binding site. Expression of these GMAP210 fragments did not alter Golgi structure (Figure S1B). We progressively split the JAF174 fragment into smaller pieces and tested their ability to IP IFT20 and displace IFT20 from the Golgi complex. The smallest fragment of GMAP210 that IPed IFT20 and displaced IFT20 from the Golgi contained residues 1180 to 1319 (JAF203). However, this peptide was not as effective as the slightly larger 1157 to 1319 fragment (JAF192). In all cases, the ability to IP IFT20 correlated with the ability to displace IFT20 from the Golgi complex. In contrast, the ability of the GMAP210 fragment to localize to the Golgi complex was not correlated with the presence of the IFT20 binding site. This suggests that GMAP210 localization to the Golgi complex is not dependent on IFT20. This appears to be the case as cells lacking IFT20 still localize GMAP210 to the Golgi complex (data not shown).

The amino acid sequence of the IFT20 binding domain in GMAP210 is 95% identical between humans and mice while overall the two proteins are 80% identical, suggesting that there is selective pressure maintaining the IFT20-binding sequence. The IFT20 binding site is not found in the *Caenorhabditis* or *Drosophila* GMAP210 homologues.

### Generation of a GMAP210 Mutant Mouse

**Figure 3:**
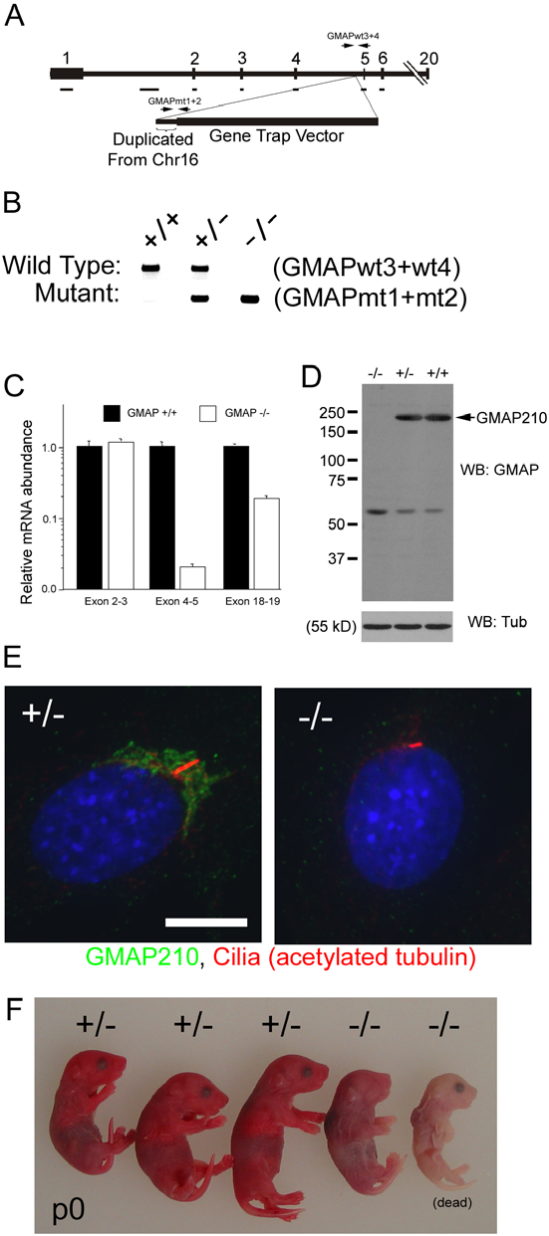
Generation of a GMAP210 mutant mouse. A. Schematic drawing of the GMAP210 AJ0290 allele. This allele contains the gene trap vector plus 531 base pairs of DNA duplicated from chromosome 16 inserted into the fourth intron. Exons are numbered above the bar. The lines under the exons and intron 1 indicate fragments of genomic DNA that were verified to be present by PCR. Positions of genotyping primers are indicated by the pairs of arrows. B. Genotyping primers (see materials and methods and diagram in A) spanning the insertion site were designed to detect the wild-type allele while primers in the insertion were used to detect the mutant allele. C. Real Time qPCR was used to measure the relative transcript levels in lung for parts of the message upstream of the insertion site (exons 2–3), at the insertion site (exons 4–5), and downstream of the insertion site (exons 18–19). GMAP data were expressed relative to GAPDH mRNA and normalized to wild-type expression for each primer pair. n = 9–10 mice/point. Note the logarithmic scale on the ordinate axis ** p<0.01, unpaired t-tests. D. Western blotting of MEF cells generated from homozygous mutant, heterozygous and homozygous wild-type embryos. Antibody was generated against the C-terminal end of the protein and detects GMAP210 and a smaller non-specific band at ∼60 kD. Tubulin was used as a loading control. E. Embryonic fibroblasts from heterozygous (+/−) and homozygous (−/−) mutant embryos stained with GMAP210 (green) and acetylated α-tubulin (red) antibodies plus DAPI (blue). Scale bar is 10 µm. F. Photo of a new born liter of animals. The fourth animal from the left was alive at the time of the photo, while the right most animal had died prior. Genotypes are above the animals.

To begin to understand the *in vivo* function of GMAP210, we obtained mouse gene trap ES cell line AJ0490 from the Sanger Institute [40] and used these cells to generate a mutant mouse. Cell line AJ0490 contains a splice acceptor site and a β-galactosidase-neomycin resistance gene fusion inserted into intron 4 of GMAP210. There also is an insertion of 531 bp derived by duplication from chromosome 16 at the junction between the vector and intron 4 (Figure 3A). In spite of this duplication, the rest of the gene appears intact as measured by PCR of exons and selected other regions of genomic DNA (Figure 3A). Sequencing of cDNA made from the AJ0490 allele indicates that the first four GMAP210 exons are spliced to the 5′ end of the β-galactosidase message, potentially producing a fusion protein containing the first 197 residues of GMAP210 fused to the N-terminus of β-galactosidase. Real time RT-PCR of mRNA from e18.5 lungs indicates that the message derived from exons upstream of the insertion is found at about the same level as controls but significantly less message is made from the exons downstream of the insertion (Figure 3C).

Since the commercially available GMAP210 clone 15 Ab did not detect mouse GMAP210, we generated a rabbit polyclonal directed against the C-terminal tail of the mouse protein. In extracts made from wild type and heterozygous mouse cells, this antibody recognizes a band of ∼200 kD that is likely to be GMAP210 and a cross reacting band of ∼60 kD. The observation that the 200 kD band is missing in the homozygous mutants, without the presence of any new smaller bands, suggests that the downstream exons in the mutant allele are not translated significantly (Figure 3D). In addition, immunofluorescence analysis of MEFs from mutant animals did not show any staining with this antibody whereas GMAP210 was readily detected at the Golgi complex in the control MEFs (Figure 3E). This data suggests that the AJ0490 allele is either null or a strong hypomorph.

**Figure 4:**
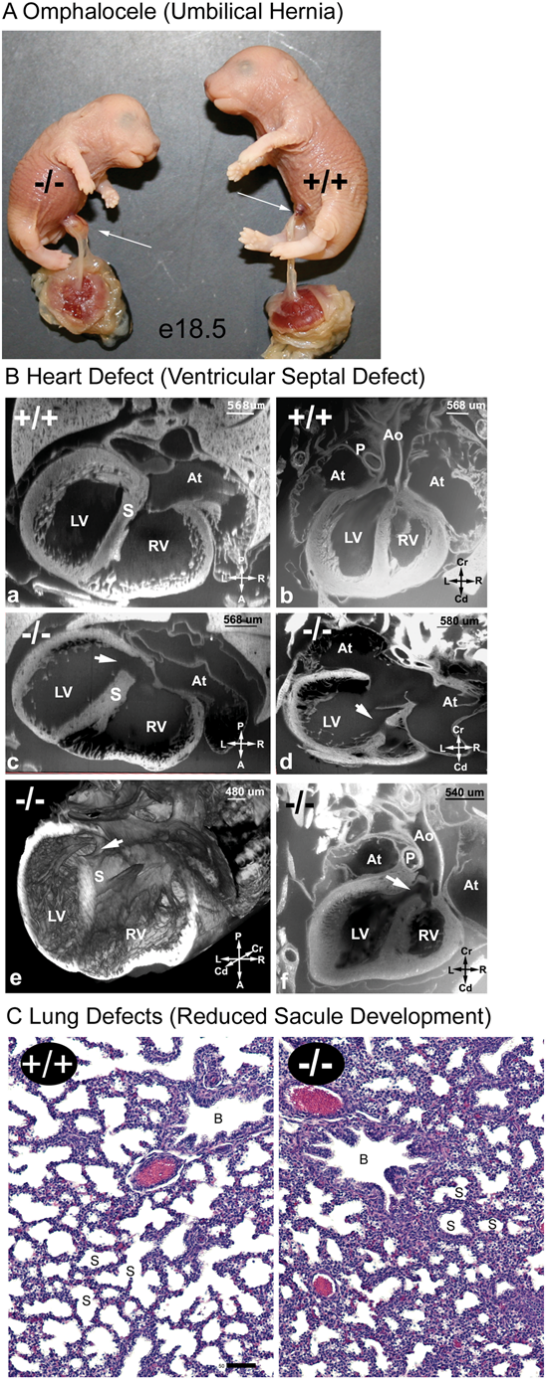
Characterization of the GMAP210 mutant mouse phenotype. A. Gross Morphology of GMAP210 Mutants. Image of a pair of e18.5 embryos. Note the open mouth and omphalocele (arrow) on the mutant embryo. B. Cardiovascular defects in the GMAP210 Mutants. (Ba-Bb, wild type) EFIC images of a wild type e18.5 embryo showing normal septation of the ventricular chambers (Ba), contrasting with the VSD seen in mutant embryo shown in (Bc). The aorta (Ao) connects with the left ventricle (LV) (Bb), which contrasts with the mutant embryo shown in (Bf) with an overriding aorta that connects to both ventricles via a VSD. (Bc-Be, mutant) EFIC images of a mutant embryo at e18.5. In the transverse imaging plane (Bc), a large muscular ventricular septal defect (white arrow) can be seen situated posteriorly (see Video S1). Examination in the frontal plane (Bd) showed a second muscular ventricular septal defect (white arrow) positioned anteriorly (see Video S2). Three dimensional reconstruction of the heart (Be) shows the posterior ventricular septal defects (white arrow; also see Video S3). (Bf, mutant) Another mutant fetus at E18.5 examined by EFIC imaging was found to have a ventricular septal defect (white arrow) associated with a overriding aorta (Ao) (see Video S3). Pulmonary stenosis was indicated with a reduction in the size of the pulmonary outflow (P) compared to the aorta (Ao). There was also apparent thickening of the ventricular chamber walls. This constellation of defects is consistent with Tetralogy of Fallot. At: atrium, LV: left ventricle, RV: right ventricle, S: septum, Cr: cranial, CD: caudal, L: left, R: right, A: anterior, P: posterior. C. Lung. Hematoxylin and eosin staining of e18.5 embryonic lungs. The larger airways are marked with B and the saccules are marked with S. Note the smaller saccule space and thicker inter saccule mesenchyme in the mutant at e18.5. Scale bar is 50 µm for e18.5 and 100 µm for e15.5.

The GMAP210 gene trap allele causes a neonate lethal phenotype as all homozygous mutant animals were found either dead or close to death on the morning of their birth and none survived past postnatal day 0 (p0) (25 +/+, 56 +/−, 14 −/− from 18 litters. +/+ and +/− were genotyped at various ages between p0 and p21, all −/− were genotyped at p0). Mutants on p0 never achieve the healthy pink color of normal littermates but rather appear cyanotic or pale bluish pink (Figure 3F). Mutants that were found alive were inactive but occasionally made a convulsive or a gasping like movement. Less than expected numbers of homozygous mutants were found but this is likely due to cannibalism of dead pups, since roughly Mendelian numbers of mutant embryos (44 +/+, 50 +/− , 33 −/− from 18 litters) were found at embryonic day 18.5 (e18.5), one day prior to birth. Mutant embryos at e18.5 were smaller than normal, weighing on average 70±9% (n = 6 litters) of what +/− and +/+ embryos weigh. In addition, the mutants usually had their mouths open with protruding tongues, suggesting craniofacial anomalies, and some exhibited an omphalocele or abdominal wall hernia, indicating a body wall closure defect (Figure 4A). The omphalocele has also been observed by D. Beier in an independently identified allele (David Beier, personal communication). We observed no evidence of polydactyly, left-right patterning defects or hydrocephaly, which are common phenotypes also associated with cilia defects.

To understand the pathology causing neonatal lethality in the mutant animals, we fixed embryos at e18.5, the day prior to birth, and examined them histologically. The abdominal organs did not appear to be greatly affected by the absence of GMAP210 and we did not detect any abnormalities in the kidney or pancreas. However in the thoracic cavity, both the heart and lungs were affected. To characterize the heart defect, 5 mutant and control animals were fixed in formalin and the hearts analyzed using episcopic fluorescence image capture (EFIC) [41]. With EFIC imaging, we generated serial 2D image stacks and 3D reconstructions that allowed detailed examination of the cardiac anatomy in multiple imaging planes (see Video S1, S2, S3). All five mutant hearts showed ventricular septal defects (VSD). Normally, at e18.5 ventricular septation is complete, allowing for separate pulmonary vs. systemic circulation from the right vs. left ventricles (Figure 4Ba,b +/+). However in the mutants, muscular VSDs are found at the anterior and posterior walls of the heart, which would cause inappropriate mixing of blood (Figure 4Bc–f −/−; see Video S1, S2, S3). In one mutant, a VSD was observed in conjunction with an overriding aorta, which is an abnormal positioning of the aorta between the right and left ventricle. This was accompanied by a narrowing of the pulmonary outflow (pulmonary stenosis) and thickening of the ventricular chamber walls (Figure 4Bf). Together this constellation of defects is consistent with Tetralogy of Fallot (Figure 4Bf), which in humans, is a relatively common but serious congenital heart condition.

The lungs showed the normal four right and one left lobe structure suggesting that the early stages of development had occurred normally. However, at e18.5 the mutant animals had notably smaller saccules with thicker inter-saccule mesenchyme (Figure 4C). Mutants had about one third as much saccule space as littermate controls (Wild type = 33.6±9%, Mutant = 12.9±5%, n = 5 animals for each genotype). At e18.5 mouse lungs are normally in the terminal sac stage of development. During this stage, which lasts from e17.5 to p5, the lung mesenchyme thins to bring the capillaries next to the prospective alveoli and the alveolar type I and II cells differentiate [42]. During their differentiation, the Type I cells flatten to reduce the distance between the capillaries and the air exchange surface of the saccule and the Type II cells produce surfactant for secretion into the saccules. Staining control and mutant lungs with markers for the Type I and II epithelial indicates that both types of cells are present. However in the mutant lungs, the Type II surfactant secreting cells are not as clearly interdigitated between the Type I cells and the SP-C staining is more punctate and distributed throughout the cell rather than being located at the apical end as it is in the control lungs (Figure S2B). Staining with PECAM1, to mark the endothelial cells of the capillaries, indicates that capillaries are forming in the mutant lungs like the wild type but in the mutant lungs the capillaries are less associated with the saccules (Figure S2A). EM analysis indicates that the type I cells are less flattened in the mutant lungs as compared to the controls (Figure S2C). Quantitative PCR was used to examine the expression levels of a number of lung development genes. Genes examined included sonic hedgehog (SHH), which is critical for branching morphogenesis, VEGF-A, which is a regulator of vascular development, Hif1a and its binding partner ARNT, which regulate transcription of VEGF-A and other genes, SP-C, which encodes a surfactant molecule critical for lung function at birth, and the selenium binding protein, SelenBP1, which is up regulated just before birth [43]. No differences were seen between the mutant and control animals (data not shown).

### Cellular Function of GMAP210

**Figure 5:**
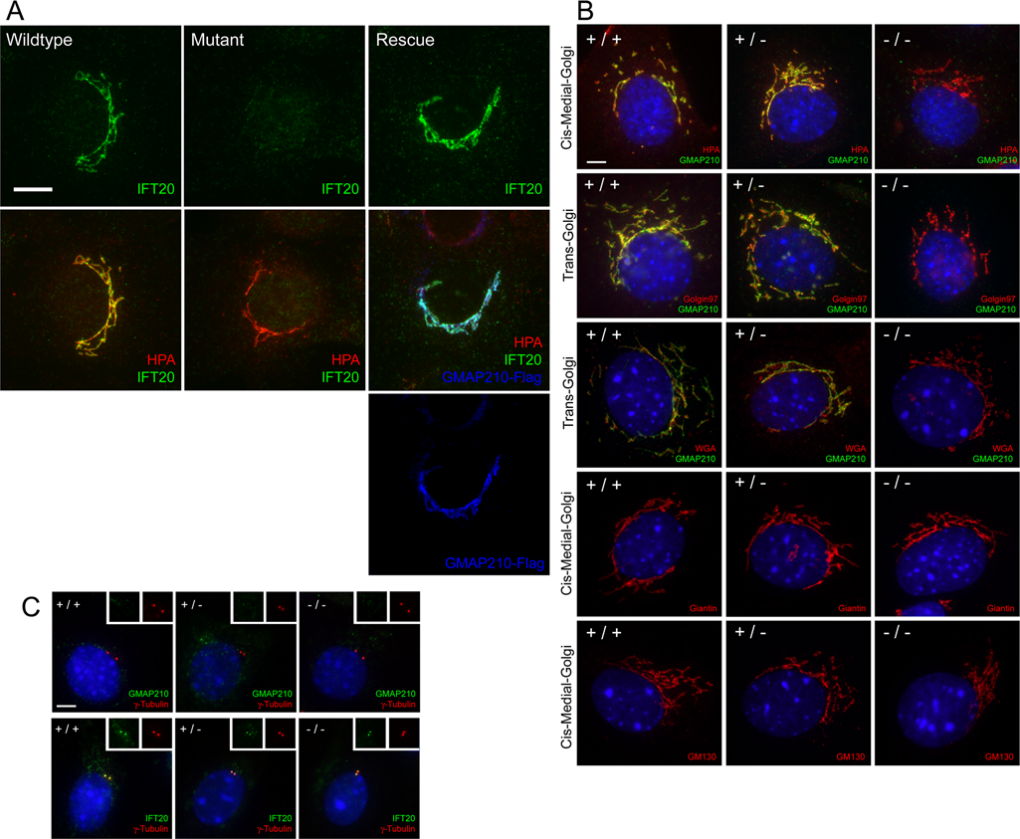
Characterization of the GMAP210 cellular phenotype. A. MEK cells labeled with IFT20 (green), the cis-medial Golgi marker HPA (red) and FLAG (blue). Scale bar is 10 µm. B. MEK cells labeled with Golgi markers HPA, Golgin97, WGA, Giantin, and GM130 (red). The Golgi compartment stained by each marker is listed on the left side of the row. Cells also are labeled with DAPI (blue) and GMAP210 (green) if the markers are compatible. Scale bar is 5 µm. C. MEK cells were fixed with methanol and labeled with DAPI (blue), the centrosome marker gamma tubulin (red), and either GMAP210 (green, top panel) or IFT20 (green, bottom panel). Insets show the green and red channels of the centrosome region. Note that the methanol fixation extracts most of the Golgi pools of IFT20 and GMAP210 (see [32]). Scale bar is 5 µm.

To begin to understand GMAP210s function in cells, we generated embryonic fibroblasts (MEFs) and kidney (MEKs) cells from e18.5 animals. All three genotypes (+/+, +/−, −/−) grew at similar rates and outwardly appeared indistinguishable. Since GMAP210 was identified as an IFT20 binding partner, we sought to understand how the lack of GMAP210 affected IFT20 and cilia formation. Wild-type MEKs and MEFs (not shown) localize IFT20 to the Golgi apparatus (Figure 5A, Wild Type) as we described earlier for other cell types [32]. However, IFT20 is completely dispersed from the Golgi complex in cells lacking GMAP210 (Figure 5A Mutant). This is not simply an indirect result caused by dispersal of the Golgi as the cis-medial and trans compartments of the Golgi complex appear normal in the GMAP210 mutant cells by *Helix pomatia* agglutinin (HPA), golgin97, wheat germ agglutinin (WGA), giantin, and GM130 staining (Figure 5B). The dispersal of IFT20 from the Golgi complex is caused by the lack of GMAP210 because re-expression of FLAG-tagged GMAP210 restores IFT20 to the Golgi complex (Figure 5A Rescue). IFT20 protein levels are slightly reduced in the mutant cells (data not shown) suggesting that some IFT20 is degraded when GMAP210 is absent with the rest being distributed throughout the cell. In addition to being localized to the Golgi complex, IFT20 is also found at the centrosome [32]. We were unable to detect GMAP210 at the centrosome in either the mutant or wild-type cells (Figure 5C top row) and consistent with this observation, the centrosomal pool of IFT20 is not affected in the GMAP210 mutant cells (Figure 5C bottom row). This suggests that IFT20 is not required to be trafficked through the Golgi complex in order to be assembled into an IFT particle.

**Figure 6:**
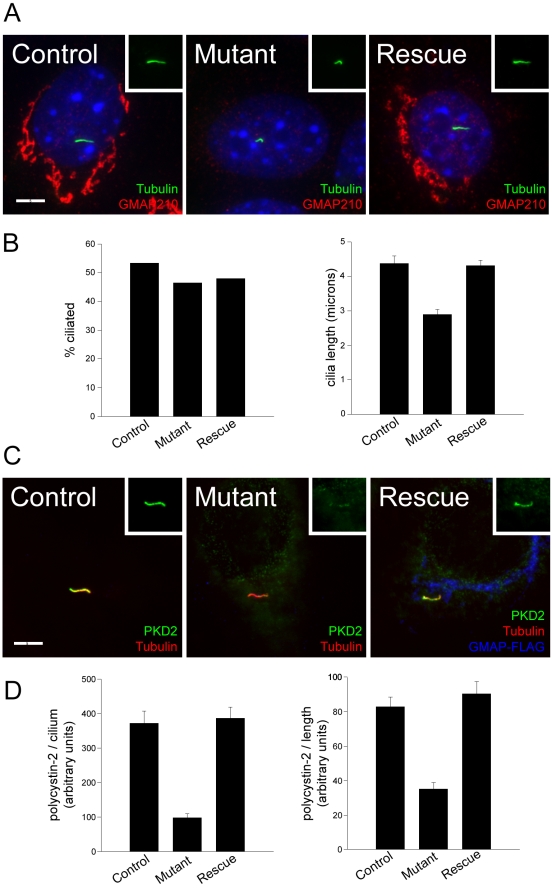
GMAP210 mutant cilia. A. MEK cells labeled with GMAP210 (red), the acetylated tubulin marker 611β1 to mark cilia (green) and DAPI (blue). Scale bar is 5 µm. Insets show the acetylated tubulin channel. B. Extent of ciliation (left panel) and cilia length (right panel) of the MEK cells after 48 hrs of serum starvation. n = 50 cilia for each genotype. C. MEK cells labeled with polycystin-2 (PKD2, green), the acetylated tubulin marker 611β1 to mark cilia (red) and FLAG (blue). Scale bar is 5 µm. Insets show the polycystin-2 channel. D. Quantification of ciliary polycystin-2 levels in control, mutant and rescued MEK cells. Polycystin-2 data are plotted per cilium on the left and corrected for length differences on the right. Error bars represent standard error of the mean (n = 46–50 cilia measured for each line). ** p<0.01.

We previously showed that IFT20 is required for ciliary assembly [32]. GMAP210 in contrast, is not absolutely required for cilia assembly (Figure 3E, 6A, Figure S2D). Quantification showed that GMAP210 mutant cells are ciliated nearly as well as control cells, and the level of ciliation did not increase upon re-expression of FLAG-tagged GMAP210 (Figure 6B, left panel). However we did note that the cilia on the GMAP210 mutant cells were often shorter than those on control cells. Measurement of cilia length on MEK cells indicated that the cilia are only about 2/3 as long as cilia on control cells. The length difference can be restored by expression of FLAG-tagged GMAP210 indicating that this result is specifically caused by the lack of GMAP210 (Figure 6B, right panel). This suggests that GMAP210 is involved in ciliary assembly.

One of the proposed roles of GMAP210 is in ER to Golgi transport [44] and there is clear evidence in yeast for the involvement of the homologue Rud3p and Rud3p-interacting proteins in membrane protein transport [45],[46]. Partial knockdown of IFT20 by RNAi reduced the amount of the membrane protein polycystin-2 on cilia suggesting that the Golgi pool of IFT20 was important for transport or retention of polycystin-2 in cilia [32]. To test the involvement of GMAP210 in ciliary transport, we measured the ciliary levels of polycystin-2 in wild-type and mutant MEKs (Figure 6C, D). The level of polycystin-2 in the mutant cilia was reduced to about one quarter the amount seen in the control line. The results are also displayed as polycystin-2 per unit of ciliary length to show that this is not an indirect effect of having shorter cilia on the mutant cells (Figure 6D right panel). To test if the decrease in ciliary polycystin-2 was specifically due to the lack of GMAP210, we transfected in FLAG-tagged GMAP210 and measured the levels of ciliary polycystin-2 in the rescued cells. Rescue with FLAG-tagged GMAP210 was able to restore ciliary polycystin-2 levels to wild type levels (Figure 6C, D). These results indicate that GMAP210 is important for efficient targeting of polycystin-2 to the cilium.

## Discussion

IFT20 is unique among the known IFT particle proteins in that it is the only one shown to localize to the Golgi complex in addition to the basal body and cilium, where the other IFT particle proteins are found. As such, it is in a unique position to coordinate the sorting or transport of ciliary membrane proteins. In prior work, we showed that an RNAi-mediated reduction of IFT20 that depleted the Golgi pool but did not greatly affect the basal body pool was sufficient to block ciliary assembly suggesting that the Golgi pool of IFT20 played an important role in ciliary assembly. We also showed that cells with a moderate reduction of IFT20 could still assemble cilia, but these cilia had less polycystin-2 in them supporting a role for IFT20 in the sorting or transport of ciliary membrane proteins [32]. In the present work, we sought to further our understanding of the function of the Golgi-associated pool of IFT20 by identifying proteins that interact with IFT20 at the Golgi complex. This analysis identified a protein called GMAP210 that binds to IFT20. Cells lacking GMAP210 fail to localize IFT20 to the Golgi complex, indicating that this protein is the linker that holds IFT20 to the Golgi. These cells can still form cilia, but they are shorter than normal and have reduced amounts of polycystin-2. Mice lacking GMAP210 die at birth likely from heart and lung defects.

As discussed below, previously published *in vitro* studies have implicated GMAP210 in a wide variety of processes ranging from Golgi structure to regulating gene expression, so the ability of GMAP210 mutant embryos to progress through embryonic development was unexpected. When examined just prior to birth, the major organs, with the exception of the lungs and heart, appear fairly normal and do not show signs of cystic disease. Our finding that ciliary levels of polycystin-2 are reduced in cells derived from the GMAP210 mutant animals would suggest that these animals should develop kidney cysts. However, in other work, we found that mice lacking cilia due to a mutation in IFT20 do not show signs of cystic disease until five to ten days after birth. When the IFT20 mutant kidneys are examined at e18.5, cilia are absent but there is no sign of cysts or even dilation of the kidney tubules [57]. Thus, it is likely that if the GMAP210 mutant animals were to live for a few weeks longer they would develop cystic kidney disease, but instead, animals die at birth before cysts have time to develop within the kidneys.

GMAP210 mutants exhibit serious congenital heart defects (VSD and Tetralogy of Fallot) that are a likely cause of the neonatal lethality observed in these animals. These disorders are common in humans, where it is estimated that as many as 1% of live births have congenital heart defects with VSDs being the most common form [47]. Tetralogy of Fallot accounts for 10% of human congenital heart disease and is the leading cause of cyanotic congenital heart disease in newborns [48]. VSDs and malalignment of the great arteries also are observed in mice with mutations in *Vangl2*, *Dvl2*, and *Scrib*. These genes are members of the planar cell polarity pathway (PCP), which regulates cell polarity and polarized cell movement via non canonical Wnt signaling [49]–[51]; for review see [52]. It is thought that formation of the ventricular septum is mediated by compaction of the trabeculae, with growth of the muscular septum generated by addition of sheets of trabeculae [53]. Cre mediated cell lineage tracing indicates the ventricular septum is derived from cells originating from the ventral aspect of the primitive ventricle, with closure of the ventricular foramen mediated by dorsal migration of this precursor cell population [54]. These cell migration events could be regulated by PCP signaling and thus VSDs might arise in animals with defects in PCP components. Similarly, the outflow tract alignment defects in the *Vangl2* mutant hearts may involve inhibition of polarized cell migration associated with myocardialization of the outflow tract. In the *Scrib* mutants, PCP defects are suggested to cause abnormalities of cardiomyocyte organization, which may result in abnormal trabeculation and ventricular noncompaction [51]. Cilia mutants often show defects in PCP [55]–[57]. For example, the deletion of IFT20 in the kidney collecting duct disrupts PCP by randomizing the orientation of the cell division plane [57]. These observations suggest cardiac defects in the GMAP210 mutants could arise from dysregulated PCP signaling due to defects in the cilia. Cilia are present in the developing mouse heart from e9.5 onward and defects in ciliary assembly cause severe heart development defects including malformation of the trabeculae that normally contribute to the formation of the ventricular septum [58]. In the GMAP210 mouse, the cilia are not absent but are likely missing key sensory receptors, analogous to the reduction of polycystin-2 in the kidney cilia (Figure 6).

The lung phenotype of the GMAP210 mutant mouse is similar to several mouse models of infantile respiratory distress syndrome caused by defects in signaling between cells of the developing lungs such as the Wnt5a [59] and the nitric oxide synthase (eNOS) mutant [60]. The Wnt5a mutant mouse is neonatal lethal and shares a number of features with the GMAP210 mutant mouse, including the thickened mesenchyme, reduced saccule space and a failure to organize its capillaries under the saccule epithelium. Wnt5a is a secreted ligand thought to be produced by both the mesenchyme and epithelial cells of the lung to regulate lung development [59]. It is of significance to note that Wnt5a mediates non canonical Wnt signaling in the PCP pathway. Thus the observed disorganization of the alveoli may reflect a disturbance of PCP signaling related to ciliary dysfunction. Similarly, the eNOS mouse is neonatal lethal and has reduced saccule space with thickened mesenchyme. In this case, it is thought that signaling between the endothelial and epithelial cells via eNOS influences development of the lung [60]. Recent studies suggest NO production in endothelial cells is regulated by shear stress transduced through the cilia, with polycystin-1 cleavage associated with loss of responsiveness to high shear stress [61]. Thus it is also possible that abnormal regulation of NO production due to ciliary abnormalities in the GMAP210 mutant may play a role in the lung defects. Based on the reduced amount of polycystin-2 in the kidney cilia, we speculate that the cilia present in the developing lung may be deficient in membrane-localized receptors and hence unable to respond to cues from the environment.

GMAP210 is a member of the golgin family of proteins. Golgins are thought to function in the formation of the Golgi matrix, which organizes the Golgi membranes and regulates membrane trafficking. Members of this family typically have large coiled-coil regions and GRIP and GRAB domains that bind to small GTPases in the ARF and ARL subfamilies [62]. In addition, GMAP210 contains an ALPS domain, which is an amphipathic helix that binds preferentially to curved membranes. In GMAP210 the ALPS domain is at the N-terminus and is separated from the GRIP and GRAB domains at the C-terminus by a long stretch of coiled-coil suggesting that it may be able to hold small vesicles on the end of a long tether [39]. Clear homologues of GMAP210 are found throughout the vertebrates and in organisms as distantly related as *Drosophila* and *Caenorhabditis*. The yeast orthologue is reported to be Rud3p as this protein shares the same domain structure being largely coiled-coil with Grab and Grab-associated domains [37]. However sequence identity between the yeast and mouse proteins is low (20–24%, BLAST E = 1e-9). BLAST analysis does not identify a *Chlamydomonas* homologue, but XP_001702340 is a coiled-coil protein containing Grab and Grab-associated domains and thus may be the *Chlamydomonas* orthologue. The IFT20 binding domain localizes within a 163 amino acid region of the GMAP210 coiled-coil domain. This sequence is well conserved throughout the vertebrate kingdom but is not present in the yeast, *Drosophila* or *Caenorhabditis* proteins nor is it found in the putative *Chlamydomonas* GMAP210 homologue. At this point, it is not known if IFT20 associates with the Golgi complex in *Drosophila*, *Caenorhabditis* or *Chlamydomonas* but if it does, it is likely to use a different mechanism. It is possible that the sorting mechanism of ciliary membrane proteins is fundamentally different in vertebrate cells as compared to *Caenorhabditis* or *Drosophila* since the cilia assembled by IFT in these invertebrates are found on dendrites and so ciliary trafficking requires sorting to dendrites before sorting to cilia. In vertebrates, this arrangement is found in olfactory sensory neurons but the majority of cells assemble their cilia directly from the cell body and do not require sorting to dendrites first. Dendritic sorting shares features with sorting to the basal-lateral domain [63] whereas most vertebrate cilia project from the apical surface if the cell is polarized.

The proposed functions of GMAP210 in the literature fall into disparate categories of organizing the microtubule cytoskeleton, organizing the Golgi complex, regulating gene expression, and playing roles in vesicular transport. Many of these studies have either not been repeated independently or are controversial. For example, Barr and Egerer called into question the role of GMAP210 as a microtubule associated protein involved in Golgi organization [33] and our data indicating that cells lacking GMAP210 still form normal Golgi structures further brings this result into question. The strongest data on the role of GMAP210 suggests that it plays roles in vesicular trafficking within the endomembrane system. In yeast, Rud3p, the GMAP210 homologue (with the caveats described above), was identified as a suppressor of mutations causing defective ER to Golgi transport [45]. Deletion of Rud3p causes glycosylation defects but the gene is not essential for viability [64]. Erv14p is required to localize Rud3p to the Golgi membrane [37]. Erv14 in yeast and its orthologue Cnih in *Drosophila* appear to play critical roles in polarized secretion. In yeast, Erv14 mutants retain the transmembrane protein Axl2p in the ER rather than inserting at the bud site [46]. In *Drosophila*, Cnih mutants retain the membrane protein Gurkin in the endoplasmic reticulum instead of secreting it at the anterodorsal corner of the oocyte [65]. Mammals have four Erv14/Cnih homologues but very little is known about their function. It will be interesting to learn if any of the Erv14/Cnih homologues are required for localization of mouse GMAP210 and IFT20 to the Golgi complex. In mammalian cells, overexpression of GMAP210 blocked the secretion of alkaline phosphatase into the medium and inhibited the retrograde transport of a KDEL-containing substrate from the Golgi to the ER [44].

The proposed function of GMAP210 in polarized secretion of proteins is interesting in the context of GMAP210 anchoring IFT20 to the Golgi complex and in being required for localization of polycystin-2 to cilia. Polarized secretion at the bud site in yeast and at the base of the cilium in other eukaryotes may be evolutionarily related and share common components. It has been proposed that the entire IFT process evolved from the coated vesicle transport system [66]. Whether this is true remains to be determined. However, it is likely that transport of membrane proteins to the ciliary membrane evolved as a specialized form of transport to the apical plasma membrane. We proposed earlier that IFT20 may function to mark vesicles that are destined for the ciliary membrane [32]. The unique ability of GMAP210 to bind IFT20 and anchor it to the Golgi membrane in addition to its ability to bind curved membranes [39] puts GMAP210 in a position to play a key role in sorting proteins to the ciliary membrane.

## Materials and Methods

### Mammalian Cell Culture

IMCD3 (ATCC) and hTERT-RPE cells (Clontech, Palo Alto, CA) were grown in 47.5% DMEM (high glucose for IMCD3, low glucose for hTERT-RPE), 47.5% F12, 5% fetal bovine serum, with penicillin and streptomycin at 37° C in 5% CO_2_. Cells were transfected by electroporation (Biorad, Hercules CA). Stable cell lines were selected by supplementing the medium with 400 µg/ml of G418 (Sigma, St. Louis, MO). Clonal lines were selected by dilution cloning after drug selection.

Primary mouse embryonic fibroblasts (MEF) were generated by dispersing e18.5 embryos in trypsin then plating in 45% DMEM (high glucose), 45% F12, 10% fetal bovine serum, with penicillin and streptomycin. Mouse embryonic kidney (MEK) cells were made by trypsin, collagenase, and DNAse dispersion [67] of e18.5 kidneys and grown in the same medium as the MEFs. 24 hrs after the MEKs were initially plated, the medium was supplemented with 150 mM NaCl and 150 mM urea to select against fibroblasts and maintained until the fibroblasts were gone.

### Immunofluorescence Microscopy

Cells for immunofluorescence microscopy were grown, fixed, and stained as described [32] except that the paraformaldehyde fixation time was reduced to 15 min. For embryonic lung immunofluorescence, lungs from e18.5 embryos were fixed overnight at 4°C with 4% paraformaldehyde in PBS and embedded in paraffin. Sections were treated with the antibodies after antigen retrieval. Labeling of the GMAP210 and PECAM-1 antibodies was enhanced with a biotin-streptavidin layer. For electron microscopy, the lungs were fixed in 4% paraformaldehyde and 2% glutaraldehyde.

Primary antibodies used included anti-tubulins (611β1, GTU-88, Sigma, St. Louis MO), anti-FLAG (Sigma), anti-MmIFT20, anti-MmIFT52, anti-MmIFT57, anti-MmIFT88 [68], anti-MmPKD2 [7], anti-human GMAP210 (clone 15, BD Transduction Laboratories), anti-T1α (8.1.1, DSHB, Univ. Iowa), anti-PECAM1 (M-20, Santa Cruz Biotechnology), anti-SP-C (FL-197, Santa Cruz Biotechnology), anti-golgin97 (CDF4, Molecular Probes). Anti-giantin, anti-GM130 (gifts from M. Fritzler, Univ. of Calgary), Anti-MmGMAP210 was made by expressing the C-terminal end of GMAP210 in bacteria (residues 1761–1976, same fragment as in JAF157, Figure 2) as a maltose binding protein fusion and injecting into rabbits. Antibodies were affinity purified against the same fragment expressed as a glutathione S-transferase fusion. Alexa 488 conjugated *Helix pomatia* agglutinin and wheat germ agglutinin was from Molecular Probes (Eugene, OR).

Widefield images were acquired by an Orca ER camera (Hamamatsu, Bridgewater, NJ) on a Zeiss Axiovert 200 M microscope equipped with a Zeiss 100× plan-Apochromat 1.4 NA objective. Images were captured by Openlab (Improvision, Waltham, MA) and adjusted for contrast in Adobe Photoshop. If comparisons are to be made between images, the photos were taken with identical conditions and manipulated equally. For the quantification of polycystin-2 in the cilia, the length, area, and average fluorescence intensity of the cilia was measured using the measurement tools of Openlab. To determine significance of differences, data were logarithmically transformed to normalize variance, subjected to one-way analysis of variance, followed by post-hoc analysis with a Tukey-Kramer test (SuperANOVA, Abacus Concepts, Berkeley CA). Confocal images were acquired by a Nikon TE-2000E2 inverted microscope equipped with a Solamere Technology modified Yokogawa CSU10 spinning disk confocal scan head. Z-stacks were acquired at 0.5 micron intervals and converted to single planes by maximum projection with MetaMorph software. Bright field images were acquired using a Zeiss Axioskop 2 Plus equipped with an Axiocam HRC color digital camera and Axiovision 4.0 acquisition software.

### Protein Analysis

FLAG-tagged IFT20, IFT25, GMAP210, and GFP were constructed by PCR amplifying the open reading frames and inserting them into p3XFLAG-myc-CMV-26 (Sigma, St. Louis, MO). FLAG IPs were carried out on stable cell lines expressing FLAG-Tagged IFT20 (JAF134), IFT25(JAF143), GFP(JAF146) or GMAP210 (full length = JAF205, shorter fragments are listed in Figure 2). Cells were rinsed once with cold PBS and lysed in Cell Lytic M+0.1% NP40 (Sigma), 0.1% CHAPSO (BioRad), plus Complete Protease Inhibitor (Roche) at 4° Celsius. Lysates were centrifuged at 18,000 g for 10 minutes and clarified lysates were incubated with Agarose beads coupled with FLAG M2 antibody (Sigma) for one hour. FLAG beads were washed 3 times with Wash Buffer (50 mM Tris, 150 mM NaCl, pH 7.4) plus 1% NP40. Bound FLAG proteins were eluted with 200 µg/ml 3× FLAG peptide (Sigma).

### Mouse Breeding

ES cell line AJ0490 was obtained from the Sanger Center and injected into C57Bl6J blastocysts to generate chimeric mice. Chimeric mice were backcrossed to the C57Bl6J inbred line and the animals used in this study were a mix of 129 and C57Bl6 backgrounds. Embryonic ages were determined by timed mating with the day of the plug being embryonic day 0.5. Genotyping was carried out with the following primer pairs: GMAPwt3 AAACAGGAGCATTTCCGAGA+GMAPwt4 AAGACATGCGCCACTATGC (product size = 295 bp in wild type) and GMAPmt1 GGGCATCCACTTCTGTGTTT+GMAPmt2 TGTCCTCCAGTCTCCTCCAC (product size = 168 bp in mutant) (Figure 3B). Mouse work was approved by the UMMS IACUC.

### Quantification of Saccule Area

Pregnant mice were euthanized by isoflurane overdose, their uteri were removed and submerged in ice cold PBS. While remaining submerged in cold PBS, the embryos were dissected from the uteri and their chests opened. The lungs were then fixed, paraffin embedded, sectioned, stained with H&E, and photographed at 4× magnification. The percent of open space (excluding bronchioles and vasculature) was calculated using the measure particle function of ImageJ.

### mRNA Analysis

Individual lungs were dissected and frozen at −80°C in RNAlater (Qiagen Inc, Valencia, CA) until RNA was isolated with RNeasy kits (Qiagen), including on-column DNA digestion. First strand cDNA was synthesized from 1 µg of total lung RNA per mouse, using a SuperScript II First-Strand Synthesis System (Invitrogen, Carlsbad, CA) and random hexameric primers. PCR primers were designed to produce amplicons between 100–150 nucleotides in length, using the online primer3 web PCR primer tool (http://fokker.wi.mit.edu/primer3/input.htm) and the IDT Primer Express software tool (http://www.idtdna.com/Scitools/Applications/Primerquest/). PCR primers were synthesized by Integrated DNA Technologies Inc (Coralville, IA) and are listed in Table S1. Real-time qRT-PCR analysis was performed using the ABI Prism 7500 sequence detection system (Applied Biosystems, Foster City, CA). Each reaction contained 2.5 ng first strand cDNA, 0.1 µM each specific forward and reverse primers and 1× Power SYBR Green (Applied Biosystems, Foster City, CA) in a 15 µl volume.

### Accession Numbers

Mouse IFT20 = NM_018854, Mouse GMAP210/TRIP11 =  XM_001001171.

## Supporting Information

Figure S1 Golgi binding site in GMAP210. A. Selected images to illustrate the Golgi binding site in GMAP210. See Figure 2A for schematic drawing of the constructs and summary of data. GMAP210 fragments were detected with FLAG antibody staining (green), endogenous IFT20 with our antibody (red) and nuclei with DAPI (blue). Note that both the N- (JAF172) and C- (JAF157) terminal ends of GMAP210 bound to the Golgi. Splitting the N-terminal fragment into two halves separated the N-terminal Golgi binding site (in JAF175) from the IFT20 binding site (in JAF174). Scale bar is 10 µm. B. Selected images to show that expression of FLAG-tagged GMAP210 fragments does not disperse the Golgi complex. Cells expressing the N-terminal coiled-coil domain (JAF172), the C-terminal grab domain (JAF157) and the IFT20 binding domain (JAF192) stained with DAPI (blue), FLAG (green), and Giantin (top row), HPA (second row), GM130 (third row) or WGA (fourth row). Scale bar is 10 µm. Note that Golgi complex is still organized in ribbons when these constructs are expressed.
(10.03 MB TIF)

Figure S2 Lung Cell Types. A. PECAM1 (green, arrow) and T1α (red, arrowhead) staining of e18.5 embryos. * mark selected red blood cells. Scale bar is 20 µm. B. Surfactant C (SP-C, green, arrow) and T1α (red, arrowhead) staining of e18.5 embryos. Blue is DAPI plus autofluorescence. * mark selected red blood cells. Scale bar is 20 µm. C. Transmission EM of e18.5 lungs. Type II cells are marked with arrows. Glycogen is marked with g. Scale bar is 10 µm. D. IFT88 (green) and 611β1 (red) staining of multi-ciliated cells in e18.5 lungs. Blue is DAPI plus autofluorescence. Scale bar is 20 µM.
(8.95 MB TIF)

Table S1 Primers used for PCR. Amplicon is the size is of the amplified product. Tm is melting temperature.
(0.04 MB DOC)

Video S1 Three dimensional reconstruction of GMAP210 mutant heart. See Figure 4Bc for details.
(8.64 MB MOV)

Video S2 Three dimensional reconstruction of GMAP210 mutant heart. See Figure 4Bd for details.
(6.90 MB MOV)

Video S3 Rotation of the three dimensional reconstruction of a GMAP210 mutant heart. The VSD can be seen at the top of the septum. See Figure 4Be for details.
(9.63 MB MOV)
