# Supplementary figures and images for: The Golgin GMAP210/TRIP11 Anchors IFT20 to the Golgi Complex
Source: PLoS Genet. 2008 Dec 26;4(12):e1000315. doi: 10.1371/journal.pgen.1000315 (PMC2602600; doi:10.1371/journal.pgen.1000315)

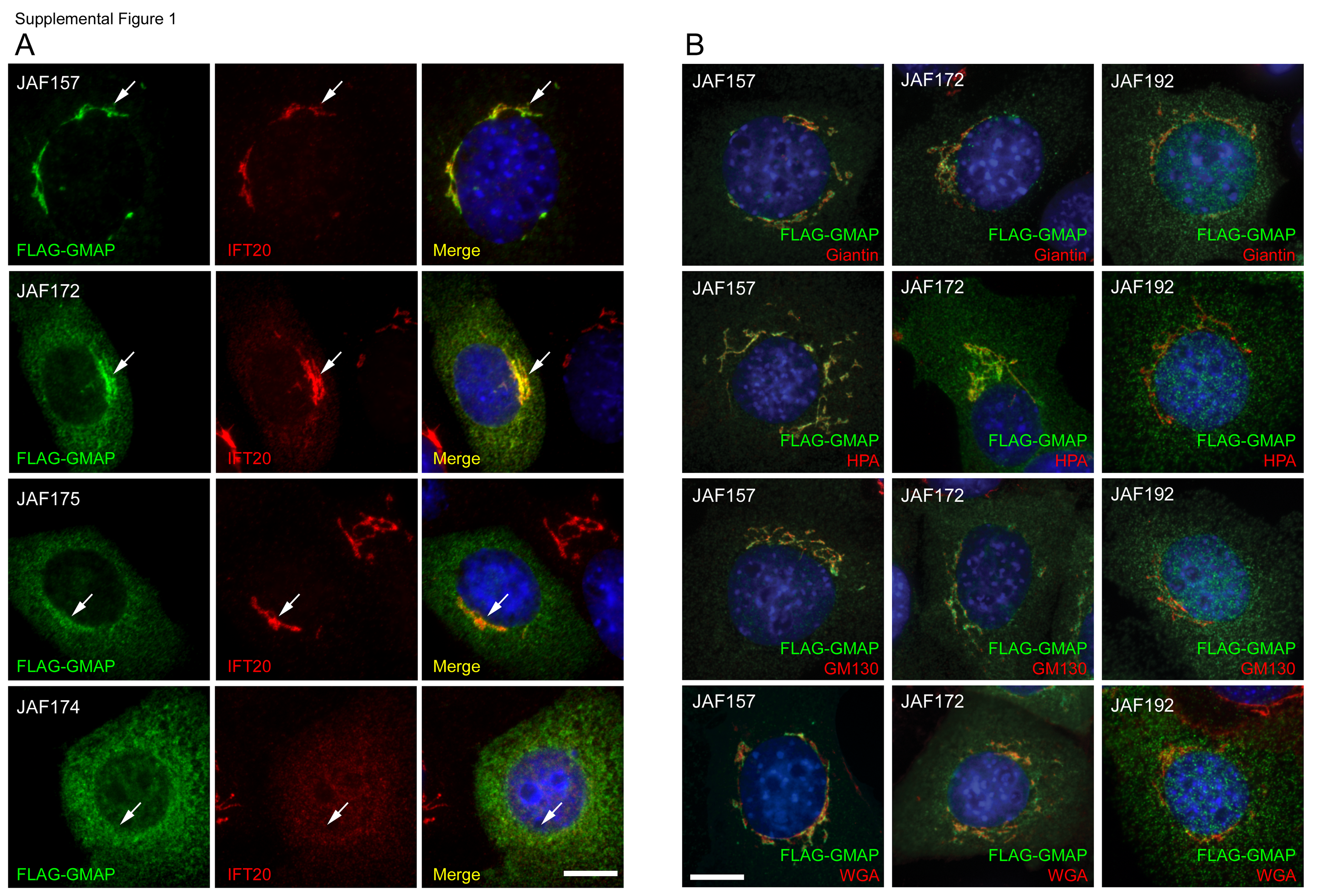

Supplement: Figure S1 — Golgi binding site in GMAP210. A. Selected images to illustrate the Golgi binding site in GMAP210. See Figure 2A for schematic drawing of the constructs and summary of data. GMAP210 fragments were detected with FLAG antibody staining (green), endogenous IFT20 with our antibody (red) and nuclei with DAPI (blue). Note that both the N- (JAF172) and C- (JAF157) terminal ends of GMAP210 bound to the Golgi. Splitting the N-terminal fragment into two halves separated the N-terminal Golgi binding site (in JAF175) from the IFT20 binding site (in JAF174). Scale bar is 10 µm. B. Selected images to show that expression of FLAG-tagged GMAP210 fragments does not disperse the Golgi complex. Cells expressing the N-terminal coiled-coil domain (JAF172), the C-terminal grab domain (JAF157) and the IFT20 binding domain (JAF192) stained with DAPI (blue), FLAG (green), and Giantin (top row), HPA (second row), GM130 (third row) or WGA (fourth row). Scale bar is 10 µm. Note that Golgi complex is still organized in ribbons when these constructs are expressed. (10.03 MB TIF) [file pgen.1000315.s001.tif]

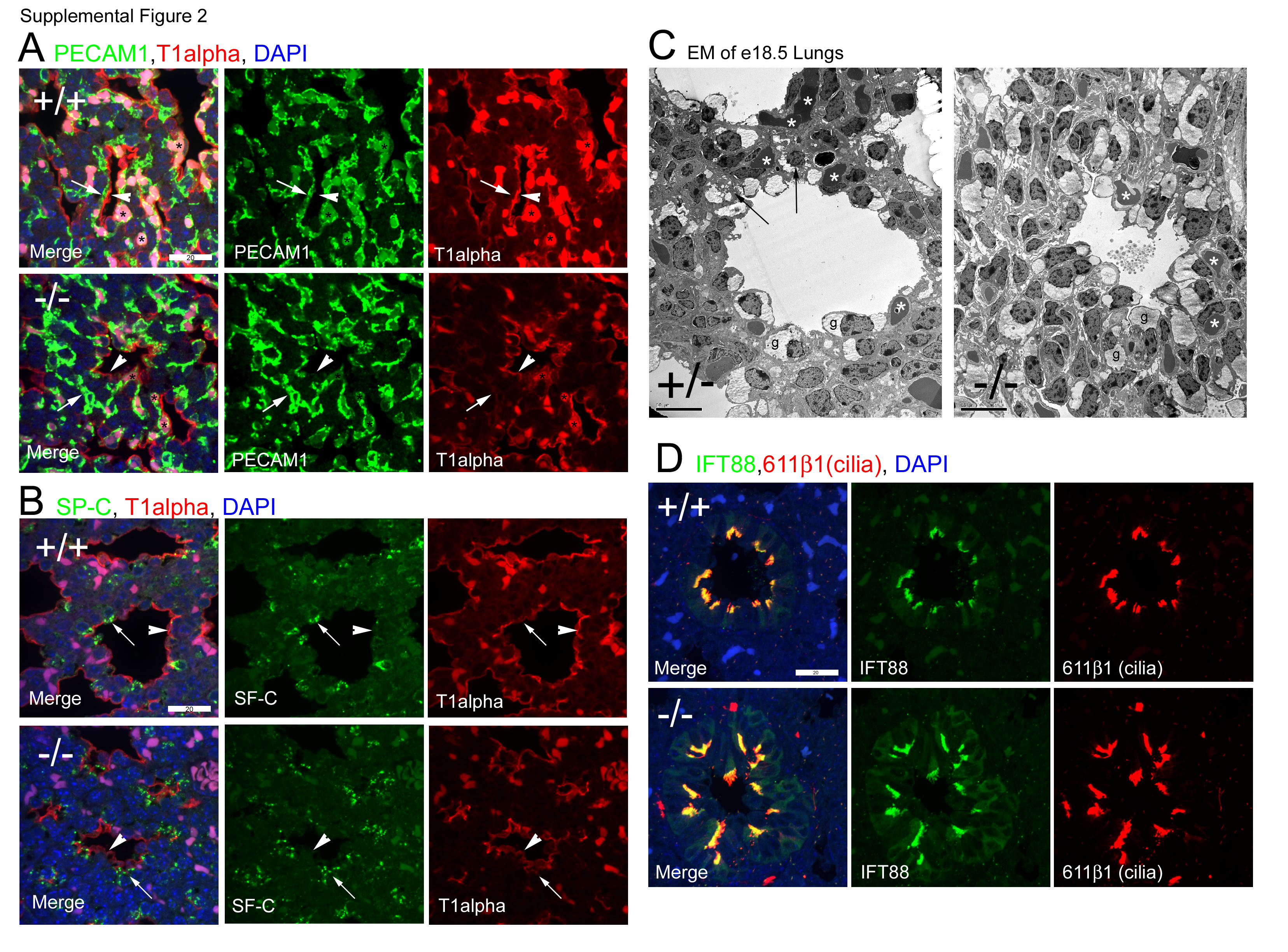

Supplement: Figure S2 — Lung Cell Types. A. PECAM1 (green, arrow) and T1α (red, arrowhead) staining of e18.5 embryos. * mark selected red blood cells. Scale bar is 20 µm. B. Surfactant C (SP-C, green, arrow) and T1α (red, arrowhead) staining of e18.5 embryos. Blue is DAPI plus autofluorescence. * mark selected red blood cells. Scale bar is 20 µm. C. Transmission EM of e18.5 lungs. Type II cells are marked with arrows. Glycogen is marked with g. Scale bar is 10 µm. D. IFT88 (green) and 611β1 (red) staining of multi-ciliated cells in e18.5 lungs. Blue is DAPI plus autofluorescence. Scale bar is 20 µM. (8.95 MB TIF) [file pgen.1000315.s002.tif]
