# Supplementary material for: The Golgin GMAP210/TRIP11 Anchors IFT20 to the Golgi Complex
Source: PLoS Genet. 2008 Dec 26;4(12):e1000315. doi: 10.1371/journal.pgen.1000315 (PMC2602600; doi:10.1371/journal.pgen.1000315)
Supplement: Table S1 — Primers used for PCR. Amplicon is the size is of the amplified product. Tm is melting temperature. (0.04 MB DOC) [file pgen.1000315.s003.doc]

### Table S1: PCR primers

| Primer | Sequence | Amplicon | Tm |
| --- | --- | --- | --- |
| MmGMAPexon4for | CCATGAAGACGACATGGACTTTGGTG | 150 | 59.7 |
| MmGMAPexon5rev | TGATCAGAGCTTTGTGCTCCCTGTACC |  | 61.9 |
| MmGMAPexon2for | TCCCTAATTCCGGGAGAGAGGAGAATG | 118 | 60.8 |
| MmGMAPexon3rev | TGAAGTTCGGATGCTTCATGCTTTTCC |  | 59.7 |
| MmGMAPexon18for | TCATGGGGAGCATCCTGGGTGT | 109 | 62.8 |
| MmGMAPexon19rev | TTCGATCCTCCTCCCAGCCATC |  | 60.7 |
| MmGAPDHExon3for | GCAATGCATCCTGCACCACCA | 138 | 61.1 |
| MmGAPDHExon4rev | TTCCAGAGGGGCCATCCACA |  | 61.1 |
| MmVEGFAexon4for | GCACAGCAGATGTGAATGCAGACC | 106 | 60.7 |
| MmVEGFAexon6rev | CCGGGATTTCTTGCGCTTTCGT |  | 60.6 |
| MmSPCexon2for | TGGTGGTCCTCGTTGTCGTGGT | 109 | 62.9 |
| MmSPCexon3rev | GTTTCCGGTGCTCCGATGCTCA |  | 62 |
| MmARNTExon4for | AGCGGTTTGCCAGGTCGGATGA | 92 | 63.5 |
| MmARNTexon6rev | CCGTCGCCGCCGTTCTATTTCA |  | 62 |
| MmSHH1exon1for | CACCCCCAATTACAACCCCGACA | 130 | 61.6 |
| MmSHH1exon2rev | TCCAGGCCACTGGTTCATCACAGA |  | 62.1 |
| MmSelBP1exon4for | TCGTGCCCCGAAGTTGCACAAG | 123 | 62.6 |
| MmSelBP1exon5rev | TCCCCCAAGGTGCTGACCATGA |  | 62.9 |
| MmHif1aExon5for | CGGGGGAGGACGATGAACATCAA | 132 | 61 |
| MmHif1aExon6rev | CACCAAGCACGTCATGGGTGGTTTC |  | 62.4 |
